# Standard: Human intestinal organoids

**DOI:** 10.1186/s13619-023-00168-5

**Published:** 2023-06-14

**Authors:** Yalong Wang, Hanqing Lin, Lianzheng Zhao, Fan Hong, Jie Hao, Zhen Zhang, Weiqi Sheng, Linhong Song, Chu-Xia Deng, Bing Zhao, Jiani Cao, Lei Wang, Liu Wang, Lingmin Liang, Wenli Kelly Chen, Chunping Yu, Zhijian Sun, Yingying Yang, Changlin Wang, Yong Zhang, Qiyuan Li, Ka Li, Aijin Ma, Tongbiao Zhao, Guoqiang Hua, Ye-Guang Chen

**Affiliations:** 1 The State Key Laboratory of Membrane Biology, Tsinghua-Peking Center for Life Sciences, School of Life Sciences, Tsinghua University, Beijing, 100084 China; 2 Guangzhou Laboratory, Guangzhou, 510005 China; 3 Guangzhou Institutes of Biomedicine and Health, Chinese Academy of Sciences, Guangzhou, 510530 China; 4 Guangzhou Hua Yi Regeneration Technology Co., Ltd, Huangpu District, Guangzhou, 510700 China; 5 D1Med Technology (Shanghai) Inc, Shanghai, 201802 China; 6 State Key Laboratory of Stem Cell and Reproductive Biology, Institute for Stem Cell and Regeneration, Institute of Zoology, National Stem Cell Resource Center, Chinese Academy of Sciences, Beijing, 100101 China; 7 Beijing Institute for Stem Cell and Regenerative Medicine, Beijing, 100101 China; 8 University of Chinese Academy of Sciences, Beijing, 100049 China; 9 Institute of Clinical Science, Zhongshan Hospital, Fudan University, Shanghai, 200433 China; 10 Department of Radiation Oncology and Cancer Institute, Fudan University Shanghai Cancer Center Fudan University, Shanghai, 200032 China; 11 Department of Oncology, Shanghai Medical College, Fudan University, Shanghai, 200032 China; 12 Shanghai Key Laboratory of Radiation Oncology, Shanghai, 200032 China; 13 Department of Pathology, Fudan University Shanghai Cancer Center, Shanghai, 200032 China; 14 Sichuan Academy of Medical Sciences & Sichuan Provincial People’s Hospital, Chengdu, 610072 China; 15 Cancer Centre, Faculty of Health Sciences, University of Macau, Macau, 999078 SAR China; 16 State Key Laboratory of Genetic Engineering, School of Life Sciences, Fudan University, Shanghai, 200438 China; 17 China Innovation Center of Roche, Li Shi Zhen Road, Pudong, Shanghai, 201203 China; 18 Eli Lilly and Company, Pudong, Shanghai, 201203 China; 19 K2 Oncology Co., Ltd, KeChuang Street, Beijing, 100176 China; 20 Qilu Pharmaceutical Co., Ltd, Jinan, 250104 China; 21 China National Institute of Standardization, Beijing, 100191 China; 22 Chinese Society for Stem Cell Research, Shanghai, 200032 China; 23 HHLIFE Co., Inc, Shenzhen, 518040 China; 24 China National GeneBank, Shenzhen, 518000 China; 25 Beijing Technology and Business University, Beijing, 100048 China; 26 School of Basic Medicine, Jiangxi Medical College, Nanchang University, Nanchang, 330031 China

## Abstract

Organoids have attracted great interest for disease modelling, drug discovery and development, and tissue growth and homeostasis investigations. However, lack of standards for quality control has become a prominent obstacle to limit their translation into clinic and other applications. “Human intestinal organoids” is the first guideline on human intestinal organoids in China, jointly drafted and agreed by the experts from the Chinese Society for Cell Biology and its branch society: the Chinese Society for Stem Cell Research. This standard specifies terms and definitions, technical requirements, test methods, inspection rules for human intestinal organoids, which is applicable to quality control during the process of manufacturing and testing of human intestinal organoids. It was originally released by the Chinese Society for Cell Biology on 24 September 2022. We hope that the publication of this standard will guide institutional establishment, acceptance and execution of proper practical protocols and accelerate the international standardization of human intestinal organoids for applications.

**Supplementary Information:**

The online version contains supplementary material available at 10.1186/s13619-023-00168-5.

## Scope

This document specifies the ethical requirements, technical requirements, and testing methods for human intestinal organoids.

This standard applies to the production and testing of human intestinal organoids.

## Normative references

The following referenced documents are indispensable for the application of these documents. For dated references, only the edition cited applies. For undated references, the latest edition of the referenced document (including all amendments) applies.

- WS 213 Diagnosis for Hepatitis C
- WS 293 Diagnostic Criteria for HIV/AIDS
- WS 299 Diagnostic Criteria for Viral Hepatitis B
- Pharmacopoeia of the People's Republic of China (2020 Edition)
- National Guide to Clinical Laboratory Procedures

## Terms and definitions

The following terms and definitions apply to this document.

### Organoids

Three-dimensional (3D) structures that grow from stem cells or progenitor cells in vitro, consist of organ-specific cell types, and can mimic the in vivo architecture and specific function of the original tissue (Clevers 2016; Fujii and Sato 2021; Kim et al. 2020; Sato et al. 2009).

### Human intestinal organoids

Organoids that develop from human intestinal stem cells of normal tissue, which are capable of self-formation, long-term growth and renewal, and possess a variety of mature intestinal epithelial cell types (Sato and Clevers 2013; Sato et al. 2011a).

### Passage

Process of dissociating existing organoids into smaller fragments, or single cell via physical, chemical, or biological methods, and keeping them growing in vitro under the same culture conditions (Ganesh et al. 2019).

### Cryopreservation

Freezing process by which organoids are maintained at low temperature in an inactive state for maintaining cellular composition, gene expression, and functional properties.

### Thawing

Process of bringing frozen organoids from an inactive to an actively growing state.

### Intestinal stem cells

Cells that can self-renew and possess the ability to differentiate into all types of intestinal epithelial cells (Barker 2014; Barker et al. 2012; Beumer and Clevers 2021; Gehart and Clevers 2019).

### Intestinal stem cell differentiation

Process of intestinal stem cells dividing into their daughter cells, including enterocytes, goblet cells, Paneth cells, enteroendocrine cells, et al. (Beumer and Clevers 2021; Gehart and Clevers 2019).

### Transit amplifying cells (TA cells)

Cells that are initially expanded from stem cells and have high proliferative capacity, and can initially differentiate into progenitor cells and further differentiate into various types of mature intestinal epithelial cells (Beumer and Clevers 2021; Gehart and Clevers 2019).

### Enterocytes

Intestinal epithelial cells that are mainly responsible for the absorption of nutrients from the intestinal lumen, which are columnar with oval nuclei located at the base of the cells and with regularly arranged microvilli located at the luminal side (Beumer and Clevers 2021; Gehart and Clevers 2019).

### Goblet cells

Intestinal epithelial cells that secret mucus periodically, which are enlarged and cup-shaped with the filled mucus particles at the top and the nucleus at the bottom (Beumer and Clevers 2021; Gehart and Clevers 2019; Gustafsson and Johansson 2022).

### Paneth cells

Intestinal epithelial cells that usually gather at the bottom of the small intestinal crypts and intermingle with intestinal stem cells, which are conical in shape with thick eosinophilic secretory granules on the top and the round nucleus at the base (Beumer and Clevers 2021; Bevins and Salzman 2011; Clevers 2013; Gehart and Clevers 2019; Porter et al. 2002).

### Enteroendocrine cells

Intestinal epithelial cells that secret intestinal hormones in response to luminal food stimulation or pH changes, which are irregularly conical with a large number of secretory particles at the bottom (Beumer and Clevers 2021; Gehart and Clevers 2019; Gribble and Reimann 2019).

## Ethics requirements

A legal and valid informed consent shall be signed by the donor who provides the tissue to develop the organoid. The consent form includes, but not limited to, potential research and therapeutic applications under the appropriate conditions, potential commercial applications of research results, and other issues applicable.

The production and research project of human intestinal cancer organoids shall be approved by the ethics review committee.

The personal information of donors shall be protected.

## Technical requirements

### Morphology

Human intestinal organoids shall be cystic or bud-like, with a cavity in the middle and tightly contacting columnar epithelial cells on the outside under optical microscopy. The cavities and the edges of the junctions shall be clear, and the cells shall be transparent (Sato et al. 2011a; Wang et al. 2022a).

### Chromosomal karyotype

The chromosomal karyotype of human intestinal organoids shall be 46, XY or 46, XX.

### Marker genes

Marker gene expressions shall be detected in human intestinal organoids, including the stem cell marker *LGR5* (Barker et al. 2007), the goblet cell marker *MUC2* (Chang et al. 1994), the enterocyte marker *ALPI* (Tetteh et al. 2016), the enteroendocrine cell marker *CHGA* (Zeve et al. 2022), and the proliferative cell marker *MKI67* (Beumer and Clevers 2021). In addition, the Paneth cell marker *LYZ* should be detected in organoids derived from small intestine (Sato et al. 2011b; Wang et al. 2022b).

### Cell composition

Human small intestinal organoids shall contain LGR5^+^ intestinal stem cells, KI67^+^ and LGR5^−^ transit amplifying cells, ALPI^+^ enterocytes, MUC2^+^ goblet cells, LYZ^+^ Paneth cells and CHGA^+^ enteroendocrine cells, the percentage of enterocytes shall be no less than 30% (Tetteh et al. 2016; Wang et al. 2020).

Human large intestinal organoids shall contain LGR5^+^ intestinal stem cells, KI67^+^ and LGR5^−^ transit amplifying cells, ALPI^+^ enterocytes, MUC2^+^ goblet cells and CHGA^+^ enteroendocrine cells, the percentage of goblet cells shall be no less than 30% (Gustafsson and Johansson 2022; Wang et al. 2020).

### Functional parameters

Alkaline phosphatase and lysozyme should be detected in human small intestinal organoids (Sensoy and Oznurlu 2019; Wang et al. 2020).

Mucins should be detected in human large intestinal organoids (Chang et al. 1994; Walaas et al. 2023).

### Culture and growth

Human intestinal organoids derived from healthy donors shall be able to be passaged for at least 5 generations in vitro after the initial culture, during which the total cell number shall not decrease. Compared to the last generation, the passaged organoids shall have the same morphology, cell composition, karyotype and other characteristics (Sato et al. 2011a).

When organoids are passaged, the cells shall be able to reconstruct into new organoids in vitro, and maintain the capacities of self-renewal and differentiation (Sato et al. 2011a).

### Viability

The organoid viability shall be ≥ 50% after thawing, and these living organoids shall be subcultured in vitro.

### Microorganisms

Organoids shall be negative for fungi, bacteria, mycoplasma, and virus.

### Identity

The identity of organoids shall match that of the donor tissue by STR analysis (Lee et al. 2015).

## Test methods

### Morphology

Observe organoid morphology by the inverted phase contrast microscope.

### Chromosomal karyotype

The method in “Preparation and quality control of animal cells for the production of biological products” from the *Pharmacopoeia of the People's Republic of China* (2020 edition) shall be followed.

### Marker genes

The method in Appendix A shall be followed.

### Cell composition

The method in Appendix B shall be followed.

### Functional parameters

For lysozyme detection, the method in Appendix B shall be followed.

For alkaline phosphatase detection, the method in Appendix C shall be followed.

For mucin detection, the method in Appendix D shall be followed.

### Quantity

Count the organoid number, defined by a pre-defined diameter threshold, from the images taken by an inverted phase contrast microscope that is attached with a scale bar.

### Viability

Organoid viability testing shall be performed on the primary organoids and passaged organoids, and the method in Appendix E shall be followed.

### Microorganisms

#### Mycoplasma

The “3301 Mycoplasma Inspection Method” in *Pharmacopoeia of the People's Republic of China* (2020 edition) shall be followed.

#### Bacteria and Fungi

The “1101 Sterility Inspection Method” in *Pharmacopoeia of the People's Republic of China* (2020 edition) shall be followed.

#### HIV

The method in WS 293 shall be followed.

#### HBV

The method in WS 299 shall be followed.

#### HCV

The method in WS 213 shall be followed.

#### Exogenous viral factors

The “3302 Exogenous Viral Factors Inspection Method” in *Pharmacopoeia of the People's Republic* (2020 edition) of China shall be followed.

### STR

The method in Appendix F shall be followed.

## Supplementary Information

**Additional file 1.**

## Data Availability

Not applicable.

## Abbreviations

Ct: Cycle-threshold
DAPI: 4,6-Diamino-2-phenyl indole
DMSO: Dimethyl Sulfoxide
HBV: Hepatitis B Virus
HCV: Hepatitis C Virus
HIV: Human Immunodeficiency Virus
PBS: Phosphate Buffer Saline
PCR: Polymerase Chain Reaction
RNA: Ribonucleic Acid
STR: Short Tandem Repeat
