# Supplementary material for: Standard: Human intestinal organoids
Source: Cell Regen. 2023 Jun 14;12:23. doi: 10.1186/s13619-023-00168-5 (PMC10267020; doi:10.1186/s13619-023-00168-5)
Supplement: Supplementary file 1 — Additional file 1. [file 13619_2023_168_MOESM1_ESM.docx]

# APPENDIX A

**Normative Appendix: Cell Type Marker Gene Test (Real-time Fluorescence Quantitative PCR Method)**

**A.1 Instruments**

A.1.1 PCR-Cycler

A.1.2 Real-time fluorescence quantitative PCR-Cycler

**A.2 Reagents**

Unless otherwise specified, the reagents used shall be analytically pure, and the water used for testing shall be deionized water.

A.2.1 Phosphate-buffered saline (PBS): pH 7.4

A.2.2 Commercial RNA extraction kit

A.2.3 Commercial RNA reverse transcription kit

A.2.4 Commercial fluorescence quantitative PCR kit

A.2.5 qPCR primers of GAPDH and target genes

**A.3 Testing protocol**

A.3.1 Organoid sample preparation

Aspirate the medium from the cultured organoids *in vitro*. Add an equal volume of phosphate buffer (A.2.1) and then aspirate. Repeat this step.

A.3.2 Organoid RNA extraction

Perform organoid RNA extraction using the commercial RNA extraction kit (A.2.2) according to the kit instructions.

A.3.3 Organoid cDNA preparation

1 μg organoid RNA is used for organoid RNA reverse transcription using the PCR-Cycler (A.1.1) and the commercial RNA reverse transcription kit (A.2.3). Perform according to the kit and instrument instructions.

A.3.4 Gene expression determination

The organoid RNA reverse transcription product from step A.3.3 is used for real-time fluorescence quantitative test. Perform using the real-time fluorescence quantitative PCR-Cycler (A.1.2), the commercial fluorescence quantitative PCR kit (A.2.4) and related qPCR primers (A.2.5) according to the kit and instrument instructions. Determine the Ct value by referring to the detection curve, then obtain the expression value of *GAPDH* (*CtG*) and target genes (*CtM*).

A.3.5 Analysis of target gene expression

Taking *GAPDH* expression as a reference, obtain the expression level of target genes: *X = CtM/CtG*.

**A.4 Calculation and analysis**

Repeat the steps A.3.1 to A.3.5 for two more times. Calculate the expression levels of organoid target genes for three times, which are recorded as the average expression levels of organoid target genes.

**A.5 Accuracy**

The absolute difference of three independent measurements obtained under repeatability conditions shall not exceed 10% of the arithmetic mean.

# APPENDIX B

**Normative Appendix: Cell Composition Proportion Test (Immunofluorescence Staining Method)**

**B.1 Instruments**

Laser confocal microscope

**B.2 Reagents**

Unless otherwise specified, the reagents used shall be analytically pure, and the water used for testing shall be deionized water.

B.2.1 Phosphate-buffered saline (PBS): pH 7.4

B.2.2 Commercial immunofluorescence staining kit

B.2.3 Antibodies of target proteins

**B.3 Testing protocol**

B.3.1 Organoid sample preparation

Aspirate the medium from the cultured organoids *in vitro*. Add an equal volume of phosphate buffer (B.2.1) and then aspirate. Repeat this step.

B.3.2 Organoid immunofluorescence staining

Perform organoid immunofluorescence staining using the commercial immunofluorescence staining kit (B.2.2) and antibodies of target proteins (B.2.3) according to the kit instructions.

B.3.3 Observation of immunofluorescence staining positive cells

The laser confocal microscope is used for observation and photography.

B.3.4 Analysis of target protein

Calculate the number of DAPI as the total number of cells in organoids (*M*). Calculate the number of cells with positive signal of target protein (*N*). The cell proportion with positive signal of target protein is obtained as *X = N/M*.

**B.4 Calculation and Analysis**

Calculate the cell proportion with positive signal of target protein in at least 30 organoids, and the average cell proportion is recorded as the proportion of target cell type in organoids.

# APPENDIX C

**Normative Appendix: Alkaline Phosphatase Test (Alkaline Phosphatase Chromogenic Method)**

**C.1 Instruments**

Microscope

**C.2 Reagents**

Unless otherwise specified, the reagents used shall be analytically pure, and the water used for testing shall be deionized water.

C.2.1 Phosphate-buffered saline (PBS): pH 7.4

C.2.2 4% paraformaldehyde solution

C.2.3 Commercial alkaline phosphatase chromogenic kit

**C.3 Testing protocol**

C.3.1 Organoid sample preparation

Aspirate the medium from the cultured organoids *in vitro*. Add an equal volume of phosphate buffer (C.2.1) and then aspirate. Repeat this step. Add an equal volume of 4% paraformaldehyde solution (C.2.2) and put it at room temperature for 20 min.

C.3.2 Organoid alkaline phosphatase staining

Perform organoid alkaline phosphatase staining using the commercial alkaline phosphatase chromogenic kit (C.2.3) according to the kit instructions.

C.3.3 Observation by microscope

The microscope is used for observation and photography.

C.3.4 Analysis of alkaline phosphatase staining

Determine whether the expression of alkaline phosphatase can be detected in organoids according to the staining results of the commercial alkaline phosphatase chromogenic kit (C.2.3).

# APPENDIX D

**Normative Appendix: Mucin Test (Mucin Staining Method)**

**D.1 Instruments**

Microscope

**D.2 Reagents**

Unless otherwise specified, the reagents used shall be analytically pure, and the water used for testing shall be deionized water.

D.2.1 Phosphate-buffered saline (PBS): pH 7.4

D.2.2 4% paraformaldehyde solution

D.2.3 Commercial mucin staining kit

**D.3 Testing protocol**

D.3.1 Organoid sample preparation

Aspirate the medium from the cultured organoids *in vitro*. Add an equal volume of phosphate buffer (D.2.1) and then aspirate. Repeat this step. Add an equal volume of 4% paraformaldehyde solution (D.2.2) and incubate at room temperature for 20 min.

D.3.2 Organoid mucin staining

Perform organoid mucin staining using the commercial mucin staining kit (D.2.3) according to the kit instructions.

D.3.3 Observation by microscope

The microscope is used for observation and photography.

D.3.4 Analysis of mucin staining

Determine whether the expression of mucin can be detected in organoids according to the staining results of the commercial mucin staining kit (D.2.3).

# APPENDIX E

**Normative Appendix: Organoid Viability Test (Calcein-AM Staining Method)**

**E.1 Instruments**

E.1.1 Inverted microscope

E.1.2 Fluorescence microscope

**E.2 Reagents**

Unless otherwise specified, the reagents used shall be analytically pure, and the water used for testing shall be deionized water.

E.2.1 Dimethyl sulfoxide (DMSO) for cell culture

E.2.2 Phosphate-buffered saline (PBS): pH 7.4

E.2.3 Storage solution of Calcein-AM solution: 2 mmol/L in DMSO

**E.3 Testing protocol**

E.3.1 Organoid counting

Place the organoids under the microscope to observe their morphology and status. Determine whether the organoid morphology meets the requirements of 6.1 by visual observation, and count the organoids with a diameter ≥20 μm.

E.3.2 Living organoid counting

Add the Calcein-AM storage solution to the medium until the final concentration is 0.2 μmol/L, and incubate the mixture for 60 minutes at 37 ℃. Then clean the medium with Calcein-AM slowly with PBS and add fresh medium. The organoids are observed and photographed by fluorescence microscope at 490 nm excitation wavelength and 515 nm emission wavelength. Living organoids are in green with clear edges. Count the living organoids with a diameter ≥20 μm.

E.3.3 Organoid counting

Tomographically scan the organoids using a microscope and image acquisition software, with the interlayer height set to the range of 10 μm to 200 μm. Superimpose the scanned images to be a single planar map, and then count the organoids in the final map.

**E.4 Organoid viability**

Organoid viability is calculated according to equation (E.1):

*X*$\text{ }\text{=}\text{（}\text{N}\text{a}\text{live}\text{/}\text{N}\text{total}\text{）}\text{×}\text{ 100}\text{\%}$ （E.1）

In this equation:

*X* —Organoid viability*,*

*N*_alive_—Number of living organoids,

*N*_total_—Total number of organoids.

**E.5 Calculation and analysis**

Repeat the procedure twice more according to E.3, calculate the average of the three living organoid ratio results, and record it as the organoid viability.

**E.6 Accuracy**

The absolute difference between the results of three independent determinations obtained under reproducible conditions shall not exceed 10% of the arithmetic mean.

# APPENDIX F

**Normative Appendix: Organoid Authentication by STR Profiling**

**F.1 Instruments**

F.1.1 Centrifuge

F.1.2 PCR-Cycler

F.1.3 Electrophoresis apparatus

**F.2 Reagents**

F.2.1 Cell DNA extraction kit

F.2.2 STR DNA profiling kit

**F.3 Sample storage**

The samples are prepared and stored below -80 ℃.

**F.4 Testing protocol**

F.4.1 Sample preparation

The organoids are cultured in the Matrigel to a stable growth state, and then they are mechanically pipetted out of the Matrigel. The mixture is collected in a centrifugal tube, the organoids are collected by centrifugation, and the supernatant is discarded.

F.4.2 Extraction of DNA

A) Perform genomic DNA extraction from organoids and primary tumor tissues according to the instructions of the cellular DNA extraction kit.

B) Measure the absorbance of extracted DNA by UV spectrophotometer to ensure that the ratio of A260/A280 is between 1.8 and 2.0.

C) DNA volume ≥20 μL, DNA concentration ≥50 ng/μL.

F.4.3 PCR amplification

A) Perform STR DNA amplification according to standard PCR amplification methods or the commercially approved kit instructions.

B) Use sterile water as the template for PCR amplification in the negative control group; use the DNA extracted from organoid and primary tumor tissue samples as a template for PCR amplification in the sample detection group; use the DNA template for amplification in the positive control group.

C) Detect the PCR products of three groups by agarose gel electrophoresis. clear target band shall be observed in positive control but not in the negative control.

F.4.4 STR genotype

Detect PCR products by capillary electrophoresis gene analyzer and STR genetic map data are obtained. The PCR banding pattern of organoids and primary tumor tissue shall be consistent.

**F.5 Results analysis**

F.5.1 When STR alleles contain the same number of repeats, only one allele peak shall appear in the profile, when they contain different numbers of repeats, two allele peaks appear in the profile.

The test is considered valid when no allele peaks appeared in the negative control group and the positive control group is consistent with its standard genotyping data.

F.5.2 If more than two allelic peaks are present at the STR locus of the tested sample, the sample shall be determined to be cross-contaminated after repeated experiments to exclude interfering factors such as mutations in the primer binding region, provided that the test is valid.
